# Genetic and Molecular Evaluation of *SQSTM1*/p62 on the Neuropathologies of Alzheimer’s Disease

**DOI:** 10.3389/fnagi.2022.829232

**Published:** 2022-02-28

**Authors:** Wei Dong, Meng-Chao Cui, Wen-Zheng Hu, Qi Zeng, Yi-Long Wang, Wei Zhang, Yue Huang

**Affiliations:** ^1^China National Clinical Research Center for Neurological Diseases, Beijing Tiantan Hospital, Capital Medical University, Beijing, China; ^2^Department of Neurology, Beijing Tiantan Hospital, Capital Medical University, Beijing, China; ^3^Key Laboratory of Radiopharmaceuticals, Ministry of Education, College of Chemistry, Beijing Normal University, Beijing, China; ^4^Department of Pharmacology, Faculty of Medicine and Health, School of Medical Sciences, University of New South Wales, Sydney, NSW, Australia

**Keywords:** *SQSTM1*/p62, Alzheimer’s disease, genetics, biomarkers, neuropathology

## Abstract

Sequestosome 1 (*SQSTM1*)/p62 is a multifunctional scaffolding protein and plays a major role in the cellular processes of autophagy, upregulation of which has been shown in several neurodegenerative disorders, including Alzheimer’s disease (AD). To investigate its genetic effects and relationship with AD pathologies, we analyzed the genetic associations of *SQSTM1* rs4935 with the risk of AD and the levels of AD biomarkers using the AD Neuroimaging Initiative (ADNI) Database. We further analyzed the distribution pattern of p62 immunoreactivity in relation to AD pathologies in the postmortem human brain tissues from AD and non-AD controls. We found that *SQSTM1* rs4935 was not associated with the risk of AD, but its T allele was significantly associated with decreased β-amyloid (1–42) (Aβ_42_) levels in the cerebral spinal fluid (CSF) of patients with AD (β = −9.336, *p* = 0.022). In addition, p62 immunoreactivity in AD is increased, but it shows an inverse relationship to Aβ deposition. A small proportion of senile plaques show p62 positive neurites. Our results suggest that *SQSTM1*/p62 may play an important role in the progression of AD *via* associations with Aβ_42_ levels in CSF and Aβ deposition in the brain of patients with AD.

## Introduction

Alzheimer’s disease (AD) is the most common cause of dementia in the elderly, accounting for approximately 60–80% of cases with dementia. AD is a neurodegenerative disorder characterized by memory loss, cognitive deterioration, functional capacity progressive impairment, and behavioral/personality abnormalities (Scheltens et al., 2016). The main pathological characteristics of AD comprise β-amyloid (Aβ) deposition, neurofibrillary tangles (NFTs) of hyperphosphorylated tau, and neuronal destruction (Duyckaerts et al., 2019). It has been indicated that pathological changes of AD begin long before the clinical manifestation (Morris and Price, 2001). Therefore, it is important to identify the biomarkers for establishing a correct diagnosis as early as possible. In the 2018 NIA-AA research framework, AT (N) classification categorized the biomarkers into three groups: “A” refers to Aβ deposition, “T” refers to pathologic tau, and “N” refers to neurodegeneration. Diminished Aβ (1–42) (Aβ_42_), elevated phosphorylated tau (p-tau), and total tau (t-tau) in the cerebral spinal fluid (CSF) were well acknowledged as diagnostic biomarkers for AD research (Jack et al., 2018). Postmortem neuropathology examination remains the gold standard in AD diagnosis. Thal amyloid phase, Braak NFT staging, and CERAD neuritic plaque score were incorporated into the recommendations for neuropathological measurements of dementia, and the ABC scoring system was derived to assess AD-featured neuropathologies (Montine et al., 2012).

Sequestosome 1/p62 (*SQSTM1*/p62) is a multifunctional protein that contains several protein-protein interaction domains. Through these interactions, p62 involves in the regulation of various cellular processes, such as autophagy, cell differentiation, apoptosis, and immune response (Salminen et al., 2012). The ubiquitin-associated (UBA) domain structure in the C-terminal of p62 interacts with ubiquitinated proteins, which allows it to transport polyubiquitinated protein to autophagosomes for degradation (Lamark et al., 2017). Another vital domain of p62 is the light chain 3 (LC3)-interacting region (LIR), which binds directly to LC3 and recruits ubiquitinated proteins to autophagosomal degradation pathway (Salminen et al., 2012). Autophagy involves in the degradation of intracellular damaged organelles or aggregated protein, and it is a conserved cellular process for maintaining cellular homeostasis (Levine and Kroemer, 2008). Regulating the autophagy pathway has been shown to ameliorate AD symptoms (Cai et al., 2021). The role of p62 on tau protein metabolism and NFT formation has been established (Kuusisto et al., 2002; Babu et al., 2005). Deficiency in p62 involves in a complex metabolic pathway associated with tau pathology and loss of short memory (Ramesh Babu et al., 2008). However, the expression of p62 in AD brain tissues remains controversial (Du et al., 2009; Ahmed et al., 2017). In addition, the involvement of p62 in relation to Aβ pathologies in AD is not clear, although increased p62 expression leads to reduced deposition of Aβ_42_, Aβ_40_, and amyloid precursor protein (APP) in the hippocampus of the AD animal model (Deng et al., 2020).

Genetic factors have been considered to play an important part in the occurrence and development of AD. Apolipoprotein E (*ApoE*) gene was recognized as the strongest genetic risk factor for sporadic AD (Saunders et al., 1993), and sporadic AD has been divided according to *ApoE* genetic status (Xu et al., 2021). In recent years, large-scale sequencing studies, genome-wide association studies (GWAS), and their meta-analyses with large sample sizes have elucidated many susceptible loci and disease-causing pathways in sporadic AD (Andrews et al., 2020). In the common variant meta-analysis of the Flanders-Belgian and European early onset dementia cohorts, rs4935 of *SQSTM1* was reported to have a significant association with the risk of AD (Cuyvers et al., 2015). The rs72807343 was also identified as a risk single nucleotide polymorphism (SNP) for AD in a GWAS meta-analysis (Lambert et al., 2013). However, their associations with CSF biomarkers in AD have not been investigated.

This study aims to explore the effects of *SQSTM1* polymorphism on the levels of CSF biomarkers in AD. We further investigated its protein levels in association with AD pathological markers in the postmortem human brain tissues.

## Materials and Methods

### General Information

The data used in this study were obtained from the AD Neuroimaging Initiative (ADNI) database. The ADNI study was launched as a public-private partnership, led by Principal Investigator Michael W. Weiner, MD. The foremost goal of ADNI was to test whether serial magnetic resonance imaging (MRI), positron emission tomography (PET), other biological markers, and clinical and neuropsychological assessments can be combined to measure the progression of mild cognitive impairment (MCI) and early AD. ADNI was approved by the institutional review boards of all participating institutions. A written informed consent was obtained from all participants or their guardians. For more details, please refer to www.adni-info.org. The dataset used in this study was obtained from ADNI 2 and ADNI GO subgroups, which comprised 125 AD patients and 154 normal controls. Postmortem human brain tissues from five AD and five normal controls were obtained from Human Brain Bank at the China National Clinical Research Centre for Neurological Diseases and through collaboration with MCC (Zhou et al., 2018; Supplementary Table 1).

### Single Nucleotide Polymorphisms of Interest

In the two AD risk SNPs rs4935 and rs72807343 of *SQSTM1* that were previously reported, neither of them was included in ADNI 1 genetic database, and only rs4935 was included in the ADNI 2 and ADNI GO genetic database. Thus, genotyping results of rs4935 and *ApoE* were extracted from ADNI GO/2 GWAS, which was tested using the Illumina HumanOmniExpress BeadChip (730525 markers). Quality control procedures for the genetic data were performed using PLINK software version 1.9.^1^ No deviation from the Hardy-Weinberg (H-W) equilibrium was found (*p* = 0.430). The alternative allele frequency (T allele) of rs4935 is 0.541, and the minor allele frequency is 0.459.

### Cerebral Spinal Fluid Biomarker Measurements

The data of the levels of Aβ_42_, t-tau, and p-tau_181_ in CSF were also obtained from the ADNI database. The protocol of CSF AD biomarker measurements could be found in the ADNI database. Briefly, CSF samples from all enrolled subjects were collected, frozen on dry ice, and immediately transported to ADNI Biomarker Core laboratory at the University of Pennsylvania Medical Center. The samples were then thawed at room temperature (RT), mixed gently, and used for the preparation of aliquots (0.5 ml). The levels of Aβ_42_, t-tau, and p-tau_181_ in CSF were measured using the multiplex xMAP Luminex platform (Luminex Corp., Austin, TX, United States) with the INNOBIA AlzBio3 kit (Fujirebio, Ghent, Belgium). In this study, AD characteristic CSF biomarker measurements were extracted from the “UPENNBIOMK_MASTER.csv” dataset.

### Immunohistochemistry

Three consecutive serial sections from each human brain tissue were prepared for immunohistochemistry (IHC) staining of p62, Aβ, and tau. Briefly, after deparaffinization and hydration, sections were submerged in heated citrate buffer (pH = 6) for antigen retrieval. The tissues were rinsed with phosphate-buffered saline (PBS) and immersed/incubated in 3% hydrogen peroxide and PBS for 30 min at RT. The tissues were blocked with 3% bovine serum albumin (BSA) at RT for 30 min. Tissue sections were separately incubated at 4°C overnight with the primary mouse monoclonal antibodies, namely, anti-p62 (1:2,000, clone: 2C11, Abcam, ab56416), anti-Aβ (1:2,000, clone: 4G8, BioLegend, #800708), and anti-phospho-tau (1:2,500, clone: AT8, Invitrogen, AB_223647). On the following day, the sections were washed with PBS and incubated with secondary antibody (1:200, goat anti-mouse, Servicebio, GB23301) for 1 h at RT. Finally, the immunoreactive proteins were visualized by incubation with diaminobenzidine (DAB). All the slides were counterstained with hematoxylin for IHC staining. Panoramic scanning was performed after IHC staining using the CaseViewer software (3DHISTECH, Budapest, Hungary). Densitometric analysis of p62 immunostaining was performed on the average p62 mean optical density (MOD, integrated optical density/area) of six randomly selected views covering the entire cortical region of each subject using Image pro plus software (version 6.0) to evaluate p62 immunostaining intensity, which was further compared between AD and controls. In addition, six p62 immunostaining views covering cortical layers III to IV of each AD case were selected randomly and further dichotomized into three low and three high p62 intensity areas according to their MOD values. In the same areas with characteristic p62 immunoreactivities, densitometric analysis of Aβ-positive plaque was performed on the average Aβ plaque MOD using Image pro plus software (version 6.0) to evaluate Aβ immunoreactive plaque intensity, which was further compared between the areas with low and high p62 immuno-reactive intensity. The colocalization of p62 with Aβ plaques in AD was further assessed using the consecutive immunostaining sections of p62 and Aβ.

### Statistical Analysis

The SPSS (IBM SPSS version 26.0) and PLINK version 1.9 were used for statistical analysis. SPSS *t*-test and Mann-Whitney *U*-test were used to examine continuous variables (e.g., age, education year, CSF biomarker levels, the levels of p62 protein and Aβ deposition), and chi-square test was used to test differences in categorical data (e.g., gender, rs4935 genotype, and *ApoE* ε4 status). The difference in allele frequencies in rs4935 was compared between AD patients and normal controls by the logistic regression using PLINK software with age, gender, education years, and *ApoE* ε4 status corrections. The correlations between rs4935 and the levels of CSF Aβ_42_, t-tau, and p-tau_181_ in patients with AD were estimated with multiple linear regression models using PLINK software with age, gender, education years, and *ApoE* ε4 status as covariates. GraphPad Prism software (GraphPad Prism 9.0.0) was used for data visualization. As for the missing data, multiple imputations with chained equations was performed using the *mice* package in R version 4.0.5 to avoid potential bias, assuming data were missing at random (White et al., 2011). Difference with a *p*-value < 0.05 was considered to be statistically significant.

## Results

### Generic Information of This Cohort

There was no difference in demographic factors (e.g., age, gender, and education) between AD and normal controls, although *ApoE* ε4 conjugated in patients with AD (Table 1). There was a significant reduction in Aβ_42_ levels and elevated t-tau and p-tau_181_ levels in CSF of AD compared to normal controls.

**TABLE 1:** The demographic and genetic characteristics and CSF biomarker measurements of ADNI 2/GO cohort.

	AD (*N* = 125)	NC (*N* = 154)	*P-*value
Age (years)^ab^	75.7 (10.8)	73.4 (8.4)	0.180
Gender (M/F)^c^	75/50	80/74	0.178
Education (years)^de^	15.8 (2.7)	16.4 (2.5)	0.063
*ApoE*ε4 (0/1/2)^c^	37/55/33	115/35/4	<0.001
*SQSTM1* rs4935 (TT/TC/CC)^c^	39/59/27	46/73/35	0.961
CSF Aβ_42_ (pg/ml)^ab^	129.0 (33.5)	204.0 (76.5)	<0.001
CSF t-tau (pg/ml)^ab^	121.0 (75.8)	56.7 (35.4)	<0.001
CSF p-tau_181_ (pg/ml)^ab^	51.3 (30.6)	28.3 (22)	<0.001

*Aβ_42_, β-amyloid (1–42); AD, Alzheimer’s disease; ADNI, Alzheimer’s disease Neuroimaging Initiative; ApoE, apolipoprotein E; CSF, cerebrospinal fluid; M/F, male/female; NC, normal control; p-tau_181_, phosphorylated tau_181_; SQSTM1, sequestosome 1; t-tau, total tau; the data are presented as the median (interquartile range)^a^ or mean (standard deviation)^d^; p-values for continuous variables were from Mann-Whitney U-test^b^ or unpaired t-test^e^; p-value for categorical data was from chi-square test^c^.*

### Effects of rs4935 on the Risk of Alzheimer’s Disease and the Levels of Cerebral Spinal Fluid Biomarkers in Patients With Alzheimer’s Disease

There was no significant association of rs4935 T allele with the risk of AD after correction for age, gender, education year, and *ApoE* ε4 status [odds ratio (OR) = 1.121, *p* = 0.562]. However, rs4935 T allele was significantly associated with decreased levels of CSF Aβ_42_ in patients with AD (β = −9.336, *p* = 0.022), although no significant association was found between rs4935 T allele and the levels of CSF t-tau or p-tau_181_ (Table 2). In addition, *ApoE* ε4 was significantly associated with decreased levels of CSF Aβ_42_ and increased levels of CSF p-tau_181_ in patients with AD (Table 2). Later, the associations of rs4935 T allele with the risk of AD and the levels of CSF Aβ_42_ and CSF p-tau_181_ were analyzed by stratifying *ApoE* ε4 status, but there was no ε4 status preferable to rs4935 effects (Supplementary Tables 2, 3).

**TABLE 2:** Allele frequency differences of rs4935 and *ApoE* in AD CSF biomarkers in patients with AD.

Gene	SNP allele	AD biomarker	BETA	STAT	*P*-value
*SQSTM1*	rs4935 T^1^	CSF Aβ_42_	–9.336	–2.324	0.022^a^
		CSF t-tau	–2.152	–0.274	0.784
		CSF p-tau_181_	–5.082	–1.356	0.178
*ApoE*	ε4^2^	CSF Aβ_42_	–18.485	–4.578	<0.001
		CSF t-tau	10.228	1.296	0.197
		CSF p-tau_181_	16.455	4.368	<0.001

*Aβ_42_, β-amyloid (1–42); AD, Alzheimer’s disease; ApoE, apolipoprotein E; BETA, regression coefficient; CSF, cerebrospinal fluid; p-tau_181_, phosphorylated tau_181_; SNP, single nucleotide polymorphisms; SQSTM1, sequestosome 1; STAT, coefficient t-statistic; t-tau, total tau; multiple linear regression model in PLINK was used with correction for age, gender, education year, and ApoE ε4 status^1^ or with correction for age, gender, education year, and rs4935 T allele status^2^; ^a^p < 0.05.*

### The Immunostaining Intensity of p62 in Alzheimer’s Disease and Its Relationship With Senile Plaques

**FIGURE 1:**
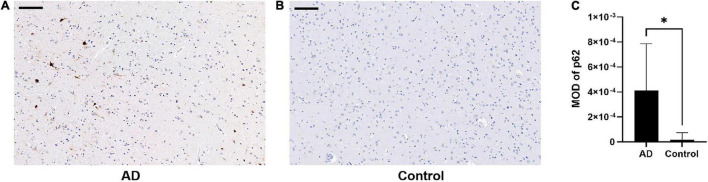
Comparison of p62 immunostaining in the temporal cortex of patients with Alzheimer’s disease (AD) and normal control. **(A,B)** There is higher p62 immunostaining intensity in AD **(A)** compared with normal control **(B)**. Scale bar: 100 μm. **(C)** Quantification of p62 immunostaining intensity based on average p62 mean optical density (MOD) in AD and normal control brains. Data were analyzed as median ± interquartile range from each subject (*n* = 5) using Mann-Whitney *U*-test. *p < 0.05.

**FIGURE 2:**
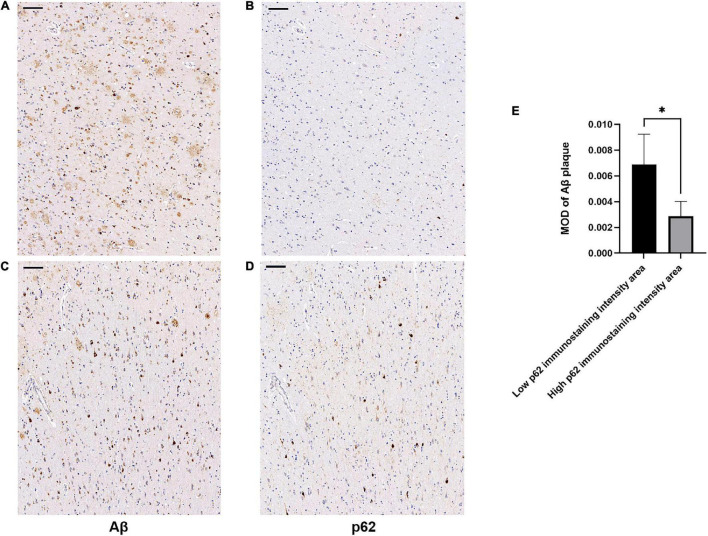
Association of β-amyloid (Aβ) and p62 immunostaining intensities in layers III to IV of temporal cortex of patients with AD. Consecutive serial sections were used for immunostaining of Aβ and p62, respectively. **(A–D)** Representative images of AD case. **(A,C)** sections were immunostained with anti-Aβ (4G8). **(B,D)** Sections were immunostained with anti-p62. There was a relatively higher density of Aβ-positive senile plaques **(A)** in the cortical area with lower p62 immunoreactive intensity **(B)**. There was a relatively lower Aβ extracellular deposition **(C)** in the area with higher intracellular p62 immunoreactive intensity **(D)**. Aβ immunostaining was also found intracellularly. Scale bar: 100 μm. **(E)** Quantification of Aβ immunostaining intensity based on average Aβ plaque MOD in low and high p62 immunoreactive intensity areas. Data were analyzed as mean ± SEM from each subject (*n* = 5) using paired *t*-test. *p < 0.05.

There was an increase in p62 immunoreactivity in patients with AD compared to controls (Mann-Whitney *U*-test, *p* = 0.032, Figure 1). Granular or fibril p62 immunopositivity was frequently found in the perikaryons, cytoplasm, axons, and dendrites of neurons, and occasionally found in the cytoplasm of glial cells. The p62 immunopositivity could also be found in the nuclear membrane. There was less NFTs density in the temporal cortical layers III to IV (Supplementary Figure 1). In layers III to IV, in the area with lower p62 immunostaining intensity, there was a relatively higher density of Aβ-positive senile plaques (Figures 2A,B), while in the area with higher intracellular p62 immunoreactive intensity, there was a relatively lower Aβ extracellular deposition, and Aβ immunostaining was also found intracellularly (Figures 2C,D). The p62 immunostaining intensity was negatively associated with the immunostaining intensity of Aβ plaque in AD human brain tissues (paired *t*-test, *p* = 0.040, Figure 2E).

**FIGURE 3:**
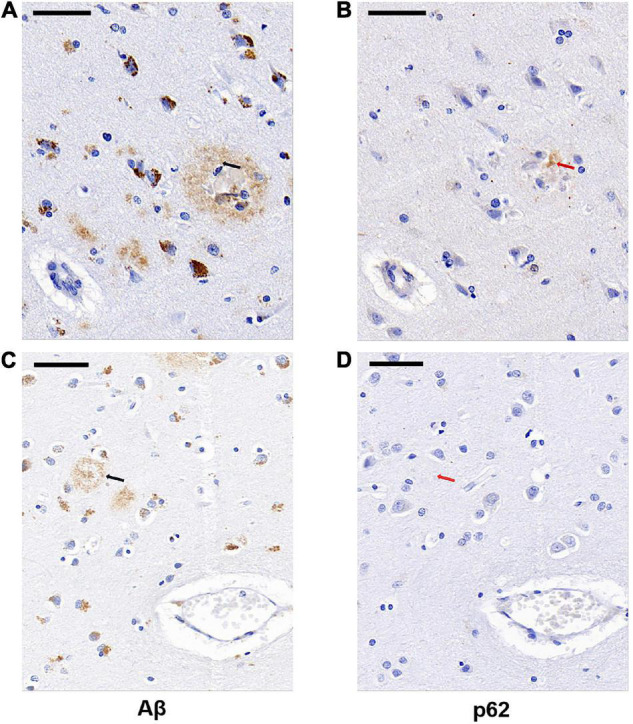
Representative images of Aβ plaque and p62 localization in AD cases. There was occasional p62-positive immunostaining (**B**, red arrowed) present in the Aβ-positive senile plaque (**A**, black arrowed). Aβ-positive senile plaque (**C**, black arrowed) did not contain p62-positive immunostaining (**D**, red arrowed). The two black arrows in **(A,C)** sections are in the same position with the two red arrows in **(B,D)** sections, respectively. Scale bar: 50 μm.

We next examined whether p62 colocalized with Aβ plaques in AD by comparing the locations of p62 and Aβ immunostainings of AD human brain tissues. We randomly selected 85 Aβ senile plaques in the temporal cortex of five AD cases. After manually matching the consecutive sections of Aβ immunostaining and p62 immunostaining, we found that p62-positive immunostaining appeared only in one Aβ-positive senile plaque, which accounted for 1.18% (Figures 3A,B). The majority of Aβ senile plaques did not contain p62 (Figures 3C,D).

## Discussion

In this study, we investigated the genetic associations of rs4935 with AD and the AD CSF biomarkers. We found that the T allele of rs4935 was not significantly associated with the risk of AD, but it was significantly associated with decreased Aβ_42_ levels in CSF of patients with AD. A lower level of CSF Aβ_42_ is a strong prognostic marker for mortality of patients with AD (Boumenir et al., 2019), which indicates that the T allele of rs4935 may lead to more severe symptoms and higher mortality in AD. In the previous study, the T allele of rs4935 significantly increased the risk of AD in the cohorts of 1,361 early onset (with early onset age and/or family disease) AD and 2,348 non-affected individuals (Cuyvers et al., 2015). The change of C to T of rs4935 is a synonymous variant identified in exon 6 (D292D). Synonymous variants may contribute to the risk of human diseases and multiple traits through affecting RNA processing, splicing efficiency, translation initiation, translation elongation, or co-translational folding (Sauna and Kimchi-Sarfaty, 2011; Rauscher and Ignatova, 2018), therein contributing to the increased risk of AD (Tang et al., 2020). The D292D is in the PEST1 domain of p62 protein. The PEST domain is enriched in proline, glutamate, serine, threonine, and aspartate. PEST sequences also serve as a proteolytic signal for rapid protein degradation relevant to short-lived proteins (Rechsteiner and Rogers, 1996). Many PEST sequences serve as targets for caspase cleavage (Belizario et al., 2008). It has been reported that p62 is a short-lived protein and a target for caspase-6 and -8, and the caspase cleavage of p62 can inhibit the autophagic process (Norman et al., 2010). T allele of rs4935 may be associated with accelerated clearance of p62 protein, leading to a jeopardized cellular process of autophagy. In AD, the monomeric Aβ peptides form oligomeric intermediates and, eventually, form amyloid fibrils and plaques. The oligomeric intermediates are tightly linked to AD pathogenesis and cause neurotoxicity (Friedrich et al., 2010; Jin et al., 2016). Since the reduced CSF Aβ_42_ level is a biomarker indicating Aβ deposition in the brain (Jack et al., 2018), the synonymous variant C to T may contribute to impaired clearance of oligomeric intermediates and, therefore, increase Aβ plaque formation due to p62 deficiency.

In this study, we found increased p62 protein intensity in the temporal cortex affected by AD compared to controls. Meanwhile, the immunostaining intensity of p62 was negatively associated with those of Aβ plaques in a certain cortical area with less NFTs density of AD, and these two molecules were barely colocalized. In the frontal cortex, the expression of p62 was reduced in AD (Du et al., 2009). However, in the temporal cortex, p62 accumulation was found in AD (Ahmed et al., 2017). Previous studies have demonstrated that p62 is involved in NFTs formation (Kuusisto et al., 2002; Babu et al., 2005). The disparity of different p62 protein levels in AD may reflect a different number of NFTs in the frontal and temporal cortices, given that NFTs are present in the temporal cortex earlier compared to the frontal cortex in AD according to Braak NFTs stages (Braak et al., 2006). This study is consistent with previous findings that p62 was absent in neuritic plaques (Kuusisto et al., 2002) and increased p62 exerted neuroprotective effects and reduced the number of senile plaques (Cecarini et al., 2020; Deng et al., 2020). The p62 accumulation in NFTs might reduce cytosolic availability of p62 and decrease its physiological function in autophagy (Feng et al., 2020), cellular signaling (Gureev et al., 2020), and protein trafficking (Ramesh Babu et al., 2008; Salminen et al., 2012). Indeed, upregulation of macroautophagy markers has been shown as an early event involved in the major pathologies, including Aβ plaques in AD (Ma et al., 2010). Our study demonstrated that in the cortex layers with no apparent involvement of NFTs, higher p62 expression may contribute to enhanced neuronal Aβ metabolism and further reduced extracellular Aβ deposition. In addition, our study also suggested that different subtypes of cortical neurons may predispose neuropathologies of AD, which requires further evaluation through molecular and morphological definition (Peng et al., 2021).

There are a couple of advantages and limitations in this study. First, we applied different techniques (genetics and pathologies) and different designs (case vs. control and case only) investigating the role of p62 in the pathogenesis of AD and had consistent findings. This study would be improved if the clinical cohort was large enough to accommodate more *SQSTM1* polymorphisms for genetic analysis and if the CSF p62 levels of patients with AD in ADNI were available for the correlation analysis with *SQSTM1* polymorphisms.

## Conclusion

The *SQSTM1* genetic variant rs4935 associates with Aβ levels in CSF of patients with AD. The p62 protein was significantly increased in AD brain tissue and its levels were negatively associated with the levels of extracellular Aβ deposition in a certain cortical area of the AD brain.

## Data Availability Statement

Publicly available datasets, which could be accessed through the ADNI website (www.adni-info.org), were used and analyzed in this study. The original data used for generating Figures 1C, 2E are given as Supplementary Tables 4, 5. The low magnification images containing Figures 2A–D are shown in Supplementary Figure 2.

## Ethics Statement

This study was approved by the Ethics Committee of Beijing Tiantan Hospital, Capital Medical University, China, and by ADNI for data analysis. Written informed consent was obtained from all participants for their participation in this study.

## Author Contributions

WD and W-ZH performed genetic analysis. WD and QZ performed neuropathological observations. WD drafted the manuscript. YH and M-CC designed the study, prepared the postmortem human brain tissues and along with WZ and Y-LW, critically revised the manuscript. All authors contributed to the article and approved the submitted version.

## Conflict of Interest

The authors declare that the research was conducted in the absence of any commercial or financial relationships that could be construed as a potential conflict of interest. The handling editor JL declared a shared parent affiliation with several of the authors WD, W-ZH, Y-LW, WZ, and YH at the time of review.

## Publisher’s Note

All claims expressed in this article are solely those of the authors and do not necessarily represent those of their affiliated organizations, or those of the publisher, the editors and the reviewers. Any product that may be evaluated in this article, or claim that may be made by its manufacturer, is not guaranteed or endorsed by the publisher.

## Footnotes

1
http://www.cog-genomics.org/plink/1.9/

## Supplementary Material

Supplementary Figure 1 Cortical distribution of neurofibrillary tangles (NFTs) in AD cases. The section was immunostained with anti-phospho-tau (1:2500, AT8, Invitrogen) and NFTs were conjugated in the super and deeper cortical layers. Scale bar: 500 μm.

Supplementary Figure 2 Representative images of Aβ plaque and p62 localization in AD. Consecutive serial sections were used for immunostaining of Aβ and p62 respectively **(A–D)**. **(A,C)** Sections were immunostained with anti-Aβ. **(B,D)** Sections were immunostained with anti-p62. High magnification of the black boxed area were shown as Figures 3A–D. Scale bar: 100 μm.
