# Supplementary figures and images for: Genetic and Molecular Evaluation of SQSTM1/p62 on the Neuropathologies of Alzheimer’s Disease
Source: Front Aging Neurosci. 2022 Feb 28;14:829232. doi: 10.3389/fnagi.2022.829232 (PMC8919032; doi:10.3389/fnagi.2022.829232)

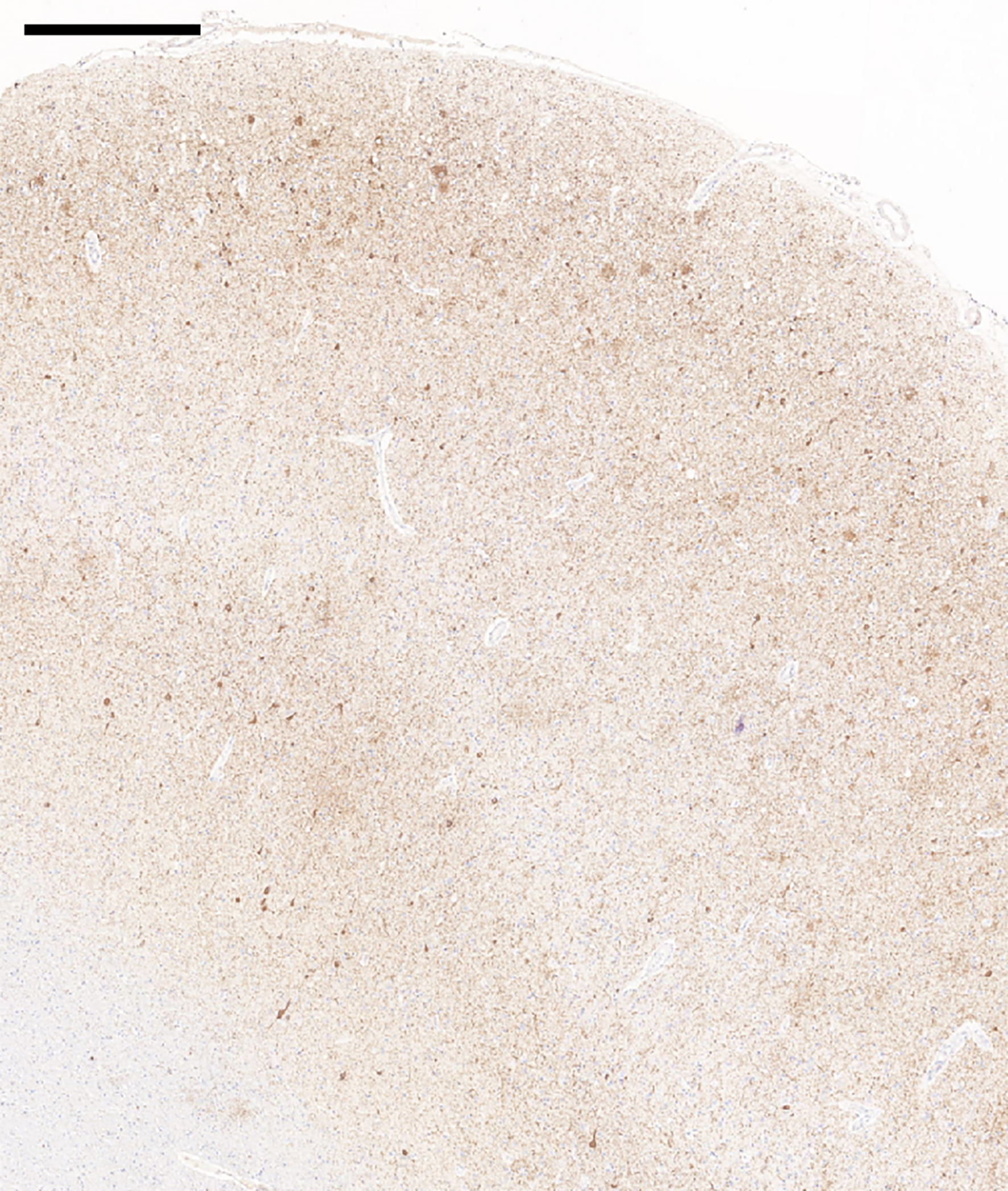

Supplement: Supplementary Figure 1 — Cortical distribution of neurofibrillary tangles (NFTs) in AD cases. The section was immunostained with anti-phospho-tau (1:2500, AT8, Invitrogen) and NFTs were conjugated in the super and deeper cortical layers. Scale bar: 500 μm. [file Image_1.TIFF]

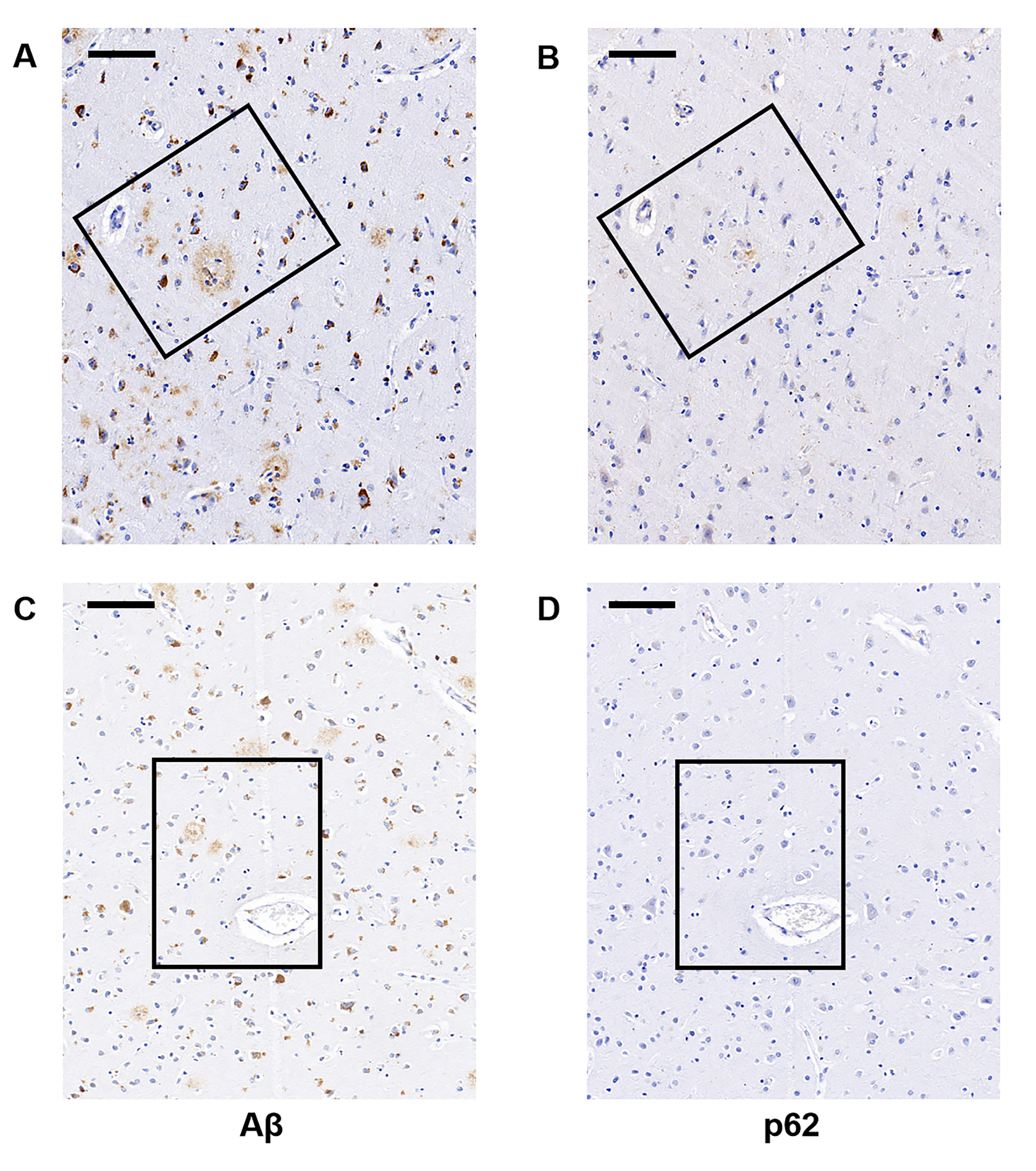

Supplement: Supplementary Figure 2 — Representative images of Aβ plaque and p62 localization in AD. Consecutive serial sections were used for immunostaining of Aβ and p62 respectively (A–D). (A,C) Sections were immunostained with anti-Aβ. (B,D) Sections were immunostained with anti-p62. High magnification of the black boxed area were shown as Figures 3A–D. Scale bar: 100 μm. [file Image_2.TIFF]
