# Supplementary material for: Genetic and Molecular Evaluation of SQSTM1/p62 on the Neuropathologies of Alzheimer’s Disease
Source: Front Aging Neurosci. 2022 Feb 28;14:829232. doi: 10.3389/fnagi.2022.829232 (PMC8919032; doi:10.3389/fnagi.2022.829232)
Supplement: Supplementary file 3 [file Table_1.DOCX]

**Supplementary table 1**. Detailed information of postmortem brain samples of

AD and control subjects

| Subjects | | Gender | Age (years) | PMI (hours) | Cause of death |
| --- | --- | --- | --- | --- | --- |
| AD | 1 | Female | 91 | 20 | NA |
|  | 2 | Male | 74 | 5 | AD |
|  | 3 | Male | 90 | 24 | AD |
|  | 4 | Female | 74 | NA | NA |
|  | 5 | Male | 84 | 5 | Severe pneumonia |
| Control | 1 | Female | 67 | 15 | NA |
|  | 2 | Female | 70 | 18 | Sepsis |
|  | 3 | Male | 76 | 7 | Myocardial infarction |
|  | 4 | Male | 85 | 8 | Cerebral stroke |
|  | 5 | Male | 70 | 7.5 | Gastric cancer |

AD, Alzheimer’s disease; NA, not available; PMI, postmortem interval.
