# Supplementary material for: Genetic and Molecular Evaluation of SQSTM1/p62 on the Neuropathologies of Alzheimer’s Disease
Source: Front Aging Neurosci. 2022 Feb 28;14:829232. doi: 10.3389/fnagi.2022.829232 (PMC8919032; doi:10.3389/fnagi.2022.829232)
Supplement: Supplementary file 4 [file Table_2.DOCX]

**Supplementary table 2.** The association between rs4935 and AD risk after stratification by *ApoE* ε4 status

|  | **rs4935 T allele+ (N)** | **rs4935 T allele- (N)** | **Total (N)** | **OR** | ***P* value** |
| --- | --- | --- | --- | --- | --- |
|  | **AD control** | **AD control** |  |  |  |
| ***ApoE* ε4+** | 69 33 | 19 6 | 127 | 0.899 | 0.704 |
| ***ApoE* ε4-** | 29 86 | 8 29 | 152 | 1.175 | 0.552 |

AD: Alzheimer’s disease; *ApoE*: apolipoprotein E; OR: odd ratio; *p* valve was calculated using the logistic regression in PLINK software, adjusted by age, gender and education year.
