# Supplementary material for: Genetic and Molecular Evaluation of SQSTM1/p62 on the Neuropathologies of Alzheimer’s Disease
Source: Front Aging Neurosci. 2022 Feb 28;14:829232. doi: 10.3389/fnagi.2022.829232 (PMC8919032; doi:10.3389/fnagi.2022.829232)
Supplement: Supplementary file 5 [file Table_3.DOCX]

**Supplementary table 3.** The associations between rs4935 and CSF Aβ_42_, CSF p-tau_181_ in AD group after stratification by *ApoE* ε4 status

|  | **rs4935** | |  | **CSF Aβ_42_** | | **CSF p-tau_181_** | |
| --- | --- | --- | --- | --- | --- | --- | --- |
|  | **T allele+ (N)** | **T allele- (N)** | **Total (N)** | **BETA** | ***P* value** | **BETA** | ***P* value** |
| ***ApoE* ε4+** | 69 | 19 | 88 | -5.892 | 0.131 | -8.017 | 0.130 |
| ***ApoE* ε4-** | 29 | 8 | 37 | -11.603 | 0.266 | -1.339 | 0.780 |

Aβ_42_, β-amyloid (1-42); AD, Alzheimer’s disease; *ApoE*, apolipoprotein E; BETA, regression coefficient; CSF, cerebrospinal fluid; *p* valve was calculated using the multiple linear regression in PLINK software, adjusted by age, gender and education year.
