# Supplementary material for: Genetic and Molecular Evaluation of SQSTM1/p62 on the Neuropathologies of Alzheimer’s Disease
Source: Front Aging Neurosci. 2022 Feb 28;14:829232. doi: 10.3389/fnagi.2022.829232 (PMC8919032; doi:10.3389/fnagi.2022.829232)
Supplement: Supplementary file 6 [file Table_4.DOCX]

| **Supplementary table 4.** The MOD of p62 immunoreactivity in AD and control groups | | | | | | | | |
| --- | --- | --- | --- | --- | --- | --- | --- | --- |
| Group | No. | MOD of p62 immunoreactivity in different microscopic fields | | | | | | |
|  |  | 1 | 2 | 3 | 4 | 5 | 6 | Average |
| AD | 001 | 8.68702E-05 | 5.24914E-05 | 5.61245E-05 | 4.88343E-05 | 4.25755E-05 | 5.87572E-05 | **5.76088E-05** |
|  | 002 | 0^a^ | 4.69058E-05 | 1.67795E-05 | 0^a^ | 1.79076E-04 | 4.19882E-05 | **4.74583E-05** |
|  | 003 | 6.28338E-04 | 9.47731E-04 | 3.52371E-04 | 5.25827E-05 | 7.90579E-05 | 4.10663E-04 | **4.11790E-04** |
|  | 004 | 7.78289E-04 | 8.59334E-04 | 2.32513E-04 | 1.29896E-03 | 8.85621E-04 | 5.79480E-04 | **7.72366E-04** |
|  | 005 | 5.20340E-04 | 8.12361E-04 | 1.41030E-03 | 1.44887E-03 | 2.80945E-04 | 3.29994E-04 | **8.00469E-04** |
| Control | 001 | 0^a^ | 0^a^ | 0^a^ | 8.87152E-05 | 1.02699E-05 | 8.44853E-06 | **1.79056E-05** |
|  | 002 | 1.46147E-05 | 1.01057E-04 | 6.2834E-05 | 0^a^ | 0^a^ | 0^a^ | **2.97509E-05** |
|  | 003 | 4.21663E-06 | 0^a^ | 0^a^ | 7.19337E-06 | 3.30619E-05 | 0^a^ | **7.41199E-06** |
|  | 004 | 2.96607E-05 | 1.07600E-04 | 1.00090E-04 | 7.37378E-05 | 9.49776E-05 | 3.14695E-04 | **1.20127E-04** |
|  | 005 | 0^a^ | 0^a^ | 0^a^ | 0^a^ | 0^a^ | 1.53961E-05 | **2.56602E-06** |

AD, Alzheimer’s disease; MOD, mean optical density; ^a^: the value was too small and approximately equal to zero indicated by Image pro plus

software; the values in bold were used for statistical calculations in figure 1C.
