# Supplementary material for: Genetic and Molecular Evaluation of SQSTM1/p62 on the Neuropathologies of Alzheimer’s Disease
Source: Front Aging Neurosci. 2022 Feb 28;14:829232. doi: 10.3389/fnagi.2022.829232 (PMC8919032; doi:10.3389/fnagi.2022.829232)
Supplement: Supplementary file 7 [file Table_5.DOCX]

**Supplementary table 5**. The MOD of Aβ plaque in the areas with low and high p62 immunoreactivity

| AD | Defining areas with low or high p62 immunoreactive intensity  Average (actual counts) | | The MOD of Aβ plaque in low and high p62 immunoreactive intensity area  Average (actual counts) | |
| --- | --- | --- | --- | --- |
|  | Low MOD of p62 immunoreactivity | High MOD of p62 immunoreactivity | Low p62 immunoreactivity | High p62 immunoreactivity |
| 001 | 5.12265E-05 (0^a^, 6.61650E-06, 1.47063E-04) | 2.89107E-04 (1.48556E-04, 2.02508E-04, 5.16256E-04) | **6.15112E-03** (4.55497E-03**,** 6.03215E-03**,** 7.86625E-03**)** | **1.12370E-03** (1.35606E-03, 1.04234E-03, 9.72698E-04**)** |
| 002 | 0 (0^a^, 0^a^, 0^a^) | 2.42613E-04 (1.26235E-04, 1.91647E-04, 4.09958E-04) | **1.19507E-03** (8.56994E-04, 1.36353E-03, 1.36468E-03) | **6.62437E-04** (1.00027E-03, 4.02903E-04, 5.84138E-04) |
| 003 | 1.06071E-04 (5.07653E-05, 1.22289E-04, 1.45158E-04) | 1.06240E-03 (8.49924E-04, 1.05644E-03, 1.28085E-03) | **2.87042E-03** (2.56255E-03, 3.16610E-03, 2.88262E-03) | **1.19739E-03** (5.85891E-05, 2.96019E-03, 5.73397E-04) |
| 004 | 3.10686E-04 (1.03407E-04, 2.19966E-04, 6.08684E-04) | 1.83779E-03 (1.53982E-03, 1.94988E-03, 2.02366E-03) | **1.04510E-02** (9.49258E-03, 7.29487E-03, 1.45657E-02) | **5.65925E-03** (5.81161E-03, 7.95951E-03, 3.20662E-03) |
| 005 | 1.87494E-04 (1.76767E-04, 1.84612E-04, 2.01102E-04) | 6.42339E-04 (2.95376E-04, 7.17456E-04, 9.14184E-04) | **1.38127E-02** (1.34747E-02, 1.03781E-02, 1.75852E-02) | **5.69182E-03** (9.02228E-03, 4.95205E-03, 3.10112E-03) |

Aβ, β-amyloid; AD, Alzheimer’s disease; MOD, mean optical density; ^a^: the value was too small and approximately equal to zero shown in Image pro

plus software; the values in bold were used for statistical calculations in figure 2E.
